# Shared-Care in Complex Malignant Hematology: An Integrative Review Using the RE-AIM Evaluation Framework

**DOI:** 10.3390/curroncol31090406

**Published:** 2024-09-14

**Authors:** Shannon M. Nixon, Dawn C. Maze, Monica Parry, Samantha J. Mayo

**Affiliations:** 1 Princess Margaret Cancer Centre, Lawrence Bloomberg Faculty of Nursing, University of Toronto, Toronto, ON M5S 1A1, Canada; samantha.mayo@utoronto.ca; 2 Princess Margaret Cancer Centre, Temerty Faculty of Medicine, University of Toronto, Toronto, ON M5S 1A1, Canada; dawn.maze@uhn.ca; 3 Lawrence Bloomberg Faculty of Nursing, University of Toronto, Toronto, ON M5S 1A1, Canada; monica.parry@utoronto.ca

**Keywords:** complex malignant hematology, shared-care, RE-AIM, implementation, travel burden, health system

## Abstract

Complex malignant hematology (CMH) shared-care programs have been established to support patients with access to care closer to home. This integrative review examined what is known about CMH shared-care using the RE-AIM evaluation framework. We searched five electronic databases for articles published until 16 January 2024. Articles were included if they were qualitative or quantitative studies, reviews or discussion papers, and reported on an experience with shared-care (defined as a reciprocal, ongoing patient-sharing relationship between a specialist centre and community hospital) for patients with hematological malignancies, and examined one or more aspects of the RE-AIM framework. The search yielded 6523 articles; 10 articles describing eight shared-care experiences. Indicators of reach were reported for 65% of the programs, and emphasized some patient eligibility criteria. Effectiveness indicators were reported for 28% of programs, and suggested favourable survival outcomes within a shared-care model; however, health system impact and quality of life studies were lacking. Indicators of adoption and implementation were reported for 56% and 42% of programs, respectively, and emphasized multidisciplinary teams, infrastructure support, and communication strategies. Maintenance was not reported. Common elements contribute to the implementation of existing CMH shared-care programs; however, a formal evaluation remains an area of need.

## 1. Introduction

Complex malignant hematology (CMH) includes acute leukemia, high-grade lymphoma, or other hematologic malignancies requiring intensive therapies, such as chemotherapy that involves prolonged hospitalization, hematopoietic stem cell transplant or chimeric antigen receptor therapy (CAR-T) [1]. This group of cancers is generally associated with rapid onset, significant symptom burden, profound cytopenias, and risk of mortality. Given the complexity of care required, CMH patients are predominantly treated in academic cancer centres with specialized facilities, concentrated resources, and highly trained multidisciplinary healthcare providers [1,2].

Travel to specialized centres may pose burdens for patients, including time toxicity and transportation expenses [3]. Patients who reside in rural locations are most vulnerable to these travel burdens [4]. Access to CMH services and quality specialized care becomes challenging, and may be a limiting factor in pursuing optimal therapy [5]. Additionally, as the CMH patient population continues to grow and age [6], there is increased pressure on specialist centres to develop innovative models of care to address capacity challenges. One way to address travel burden and capacity challenges is through expanding CMH programs by creating partnerships with community hospitals, allowing patients to receive some of their care closer to home.

Partnerships between specialized centres and community hospitals have been established in other patient populations to address geographic barriers to specialized medical care. Cohen et al. [7] reported the implementation of a shared-care model in which children with chronic conditions associated with medical fragility received complex symptom management at a community-based clinic staffed by pediatricians and nurse practitioners, affiliated with a specialist centre. Consultations with physicians at the specialist centre were available as needed. Shared-care program outcomes included enhanced patient quality of life, decreased transportation expenses, and improved healthcare utilization [7].

In the context of CMH, shared-care can enable patients to receive care on an ongoing basis from collaborative teams working in partnership across community and specialized centres. In Ontario, Canada, an acute leukemia shared-care program has been implemented, where patients receive complex care in specialist centres, and less intensive treatments and supportive care at community hospitals [8]. Hershenfeld et al. [9] evaluated this program, and found that patients who received supportive care at community hospitals within the shared-care model had reduced travel burden, and no difference in overall survival compared to patients treated at the specialist centre alone. 

Given the potential benefits of shared-care [7,9], clinicians and decision-makers would benefit from an understanding of what factors influence the successful implementation of CMH shared-care programs. The RE-AIM implementation science framework provides a guide for program evaluation, and has been widely applied to evaluate health services across a range of clinical settings and populations [10]. While other existing evaluation frameworks identify program components important for assessing implementation success [11,12,13], RE-AIM has the additional advantage of evaluating the potential for public health impact and broad program application [10]. As CMH shared-care programs may involve partnerships across specialized centres and community hospitals, it would be important to choose a framework to guide this review that addresses the potential for regional growth. This review will use the RE-AIM framework to examine what is known about the Reach, Effectiveness, Adoption, Implementation, and Maintenance of CMH shared-care [10].

## 2. Methods

An integrative literature review uses a comprehensive search strategy to provide a broad overview of current knowledge, including both qualitative and quantitative studies [14]. In January 2024, a search was conducted to identify articles reporting on CMH shared-care, which examined one or more RE-AIM framework dimensions [10]. Table 1 includes definitions used for the RE-AIM dimensions. 

The search strategy consisted of a search of the following electronic databases: Medline (1946 to 16 January 2024), EMBASE (1947 to 16 January 2024), Cochrane Database of Systematic Reviews (2005 to 16 January 2024) and Central Register of Controlled Trials (2014 to 16 January 2024), CINAHL via EBSCOhost (1974 to 16 January 2024), and Scopus via Elsevier (inception to 16 January 2024). Search terms were devised based on background reading and consultation among co-authors (SN, SM, DM, MP). Table 2 provides a sample search strategy for Medline. See Supplementary S1 for details of the search used for each database. A supplemental search of reference lists was also completed.

Articles were included if they: (a) focused on individuals of any age receiving active therapy for CMH, defined as acute leukemia, high-grade lymphoma, or other hematologic malignancies requiring intensive therapies; (b) reported on a shared-care program, defined as a reciprocal, ongoing patient-sharing relationship between levels of care (malignant hematology specialist centre and community hospital); (c) reported on at least one RE-AIM framework dimension; (d) were published as qualitative or quantitative studies, reviews or discussion papers in peer-reviewed journals; (e) reported on shared-care in a high-income country as defined by the World Bank Atlas [16], to optimize comparability to the Canadian setting; and (f) were published in English. Articles were excluded if they reported patient-sharing with primary care, office settings, or palliative care. Conference abstracts, theses, and the grey literature were excluded. 

Search results were imported to Covidence for article selection. After removing duplicates, article selection occurred through two stages of double screening. In Stage 1, two reviewers (SN and SM) independently screened titles and abstracts for relevance to the research question. In Stage 2, full texts of any relevant citations in Stage 1 were retrieved and independently reviewed by SN and SM against inclusion and exclusion criteria to determine eligibility. Any conflicts between reviewers in stages 1 or 2 were resolved through discussion until a consensus was reached. 

Extracted data from each article included the country in which shared-care was provided, the target population of the model, program details, and RE-AIM outcomes. RE-AIM outcomes were extracted using a 21-item data collection tool adapted from RE-AIM.org, which has been used in previous reviews that reported on RE-AIM dimensions [15,17]. For each RE-AIM dimension, the presence or absence of indicators was coded (yes/no), and if present, a description of the indicator was extracted. Any reported facilitators and barriers of each dimension were collected, as identified by the authors. Supplementary S2 shows all extracted indicators. SN performed all extractions, and any uncertainties were discussed with SM. 

## 3. Results

**Figure 1:**
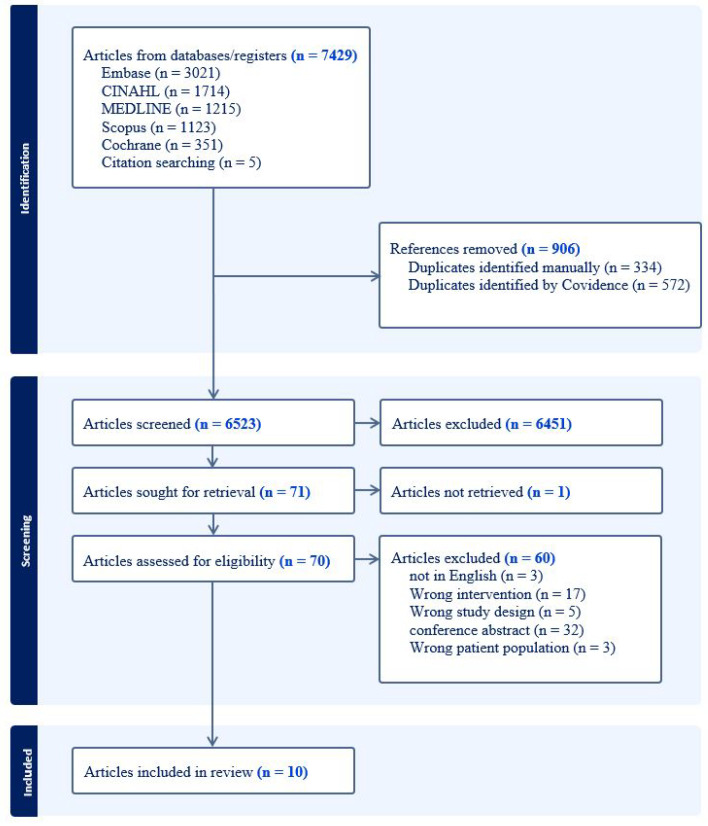
Flow Diagram of Search Strategy.

The search yielded 6523 articles after excluding duplicates. After title and abstract screening, followed by full-text screening (Figure 1), ten articles reporting on eight CMH shared-care programs were included; three papers authored by Jillella et al. [18,19,20] reported on the same CMH shared-care program and were grouped together for the purpose of analysis. All eight programs were described across observational studies (n = 4) [9,18,21,22], case series (n = 1) [23], qualitative studies (n = 1) [24], and discussion papers (n = 4) [19,20,25,26].

Programs were reported in the United States of America (n = 3) [18,19,20,21,23], Canada (n = 2) [9,25], Australia (n = 1) [24], England (n = 1) [22], and Singapore (n = 1) [26]. Programs described patients diagnosed with acute leukemia (n = 5) [9,18,19,20,21,22,23], myeloproliferative neoplasm (MPN) (n = 1) [25], lymphoma (n = 1) [26], and a variety of hematologic malignancies (n = 1) [24]. Six programs described shared-care for adult patients [9,18,19,20,21,23,25,26]; and two programs reported on the pediatric experience [22,24]. Characteristics of the included programs are listed in Table 3 and Table 4 summarizes the proportion of CMH shared-care programs reporting RE-AIM dimensions and indicators. 

### 3.1. Reach

The reach of a program considers the number and representativeness of patients willing to participate in a given initiative [10]. The methods used for selecting patients for CMH shared-care were described for most programs (6/8), though this occurred primarily from the specialist site, with the community hospital involvement not clearly addressed. For example, in a shared-care program for MPN, an intake questionnaire at the specialist centre was used to screen for disease subtypes that warrant specialist care involvement [25]. Facilitators for identifying patients for shared-care focused on strategies for enabling program referrals. For example, in Lim et al., [26] networking between hospital sites supported program awareness. In other centres, implementing standardized referral criteria facilitated the identification of appropriate patients and ensured efficiency and accuracy in referral prioritization [21,25].

The inclusion criteria for patients accepted into shared-care were described for all programs (8/8). Diagnostic criteria included subtypes of disease that would require the involvement of specialist services not available at the community hospital [18]. Treatment criteria included types of therapy that involved an aspect of care that could be safely delivered in a community setting [9]. For some programs, the inability to travel to the specialized centre determined eligibility for shared-care [23]. Exclusion criteria were described in 2/8 programs. Some patients were excluded from participating if they preferred not to receive portions of their care at a community hospital [9]. Muir et al. [22] excluded acute leukemia patients if they did not achieve remission after intensive therapy at the specialist centre and, thus, were not eligible to receive maintenance therapy at the community hospital. 

The proportion of eligible patients who were enrolled in the CMH shared-care program was reported for 1/8 programs. In Muir et al. [22], 146 patients were deemed eligible, and 59 (40%) patients participated. The rationale for declined participation was not described. In Hershenfeld et al. [9], patients were encouraged to participate in shared-care; however, it was not mandatory, and the participation rate was not reported. None of the articles provided information about the representativeness of the enrolled patients to the overall patient population in those jurisdictions (0/8). 

### 3.2. Effectiveness

The effectiveness of a program considers the measurement of primary outcomes, quality of life, and unintended negative consequences [10]. The effectiveness of the CMH shared-care programs on patient outcomes was reported by 4/8 programs. Patients receiving shared-care had improved or comparable survival outcomes compared to patients treated in specialist centres alone, or community hospitals alone [9,18,21,22]. For example, Hershenfeld et al. [9] evaluated patients with acute myeloid leukemia (AML) who received post-consolidation supportive therapy at community hospitals (n = 73), in comparison to patients who received care at the specialist centre alone (n = 344), and found no significant difference in survival between the groups: 90-day survival was 95.9% for shared-care and 95.3% for specialist centre patients. Table 5 summarizes the effectiveness data.

No programs (0/8) reported unintended consequences, such as hospital admissions, infections, and other adverse events. The impact of shared-care on hospital resources was not reported. Outcomes related to patient quality of life were reported in two programs (2/8). In the study by Hershenfeld et al. [9] mean travel distance and travel time were significantly reduced for shared-care patients travelling to their local hospitals compared to commuting from their home to the specialist centre (87.8 km and 62 min compared to 14.5 km and 18 min, respectively). In Slater et al. [24], nurses perceived that patients in the shared-care programs had less anxiety and stress; a comparator was not provided. 

### 3.3. Adoption

The adoption of a program assesses the number and representativeness of settings and staff who deliver a program [10]. All articles described some aspect of the CMH shared-care locations (8/8). Characteristics of the specialist centres included the level of healthcare provided and academic affiliation, as well as unique setting characteristics that would support specialist care. For example, specialist centres were defined as quaternary care [9], academically affiliated and a National Cancer Institute-designated cancer centre [23], and having an enhanced diagnostic pathology department for disease evaluation [25]. A description of participating community hospitals was not identified; however, Law et al. [21] described their shared-care program as part of a comprehensive, integrated healthcare delivery system comprised of 21 medical centres. 

Details of inclusion and exclusion criteria for specialist centre and community hospital participation in shared-care were identified in 2/8 programs. Goradia et al. [23] included only community hospitals with an already established partnership with the specialist centre. Hershenfeld et al. [9] included only community hospitals with transfusion and intensive care capabilities. For adoption rate, Hershenfeld et al. [9] described collaborating with 14 regional hospitals as part of their shared-care program; however, the total number of hospitals eligible for participation was not identified. Jillella et al. [18] worked with 29 community hospitals to implement shared-care; however, the number of hospitals approached for participation was not described.

The methods used to identify CMH shared-care partnerships were rarely described (3/8). In these programs, specialist centre staff identified community hospitals for participation with various communication, education, and awareness strategies. For example, in Jillella et al., [18] specialist centre physicians visited the various community hospitals to meet with stakeholders and create awareness of the need for partnerships. Jillella et al. [18] identified that strengthening relationships between specialist centres and community hospitals through on-site visits and regular meetings facilitated staff onboarding for shared-care programs. 

The characteristics of clinical staff delivering shared-care from community and specialist centres were reported for most programs (7/8), and comprised multidisciplinary team members, including physicians, nurses, nurse practitioners, clinical nurse specialists, pharmacists, social workers, and psychologists [9,18,21,23,24,25,26]. Nurse coordinator roles were implemented to facilitate shared-care and were responsible for program leadership and patient care coordination [24,25]. Community hospital staff were described as either hematologists/oncologists or community oncologists, or those with experience in managing cytopenias, chemotherapy administration, and central venous catheter care [9,18,21,23,24,25,26]. The level of staff expertise within specialist centres was less defined; however, it was described as physicians with a specialty or subspecialty in acute leukemia [23], and those with experience caring for acutely ill CMH patients [21].

Several articles reported that managing CMH patients in the community setting requires familiarity with disease-specific complications, and that establishing expertise requires time, education, and resources [18,26]. This is especially challenging when CMH patient volumes may be low [26]. It was identified that a trained, dedicated multidisciplinary team would support clinical competency, including the education of external team members such as emergency room staff [24,25,26]. Additional strategies to support the up-skilling of staff included treatment guidelines and algorithms [9,18], 24/7 live support via telephone or email for physician-physician consultation [9,18,24,26], and regular conferences and workshops to review protocols and provide updates on new treatments [9,26].

### 3.4. Implementation

The implementation of a program considers the degree to which the intervention is delivered as intended [10]. None of the articles reported on the duration of time individual patients were managed through shared-care. One program (1/8) reported the length of time patients were enrolled in shared-care, and the number of contacts with shared-care patients: a median of 3.5 in-person visits to the specialist centre over a median of 357 days of shared-care [23]. The rationale for program discontinuation for patients was not described [23]. Hershenfeld et al. [9] reported the total number of cycles of therapy that were given within the shared-care model (n = 137 cycles); however, no details were provided on length of time patients were followed on shared-care. 

While formal evaluations of fidelity were not reported, patient care was described as following pre-planned shared-care protocols (8/8). These protocols helped to ensure patients were managed in the most appropriate setting based on their medical needs over the course of treatment, while maintaining collaboration between the participating centres. In most cases, patients were admitted to the specialist centre for intensive therapy or the management of severe or infectious complications, while transferred to local community hospitals for low-dose chemotherapy and less medically intensive supportive care [9,21,22,23,24,25,26]. In some shared-care programs, community care was complemented with intermittent virtual care visits [21,23], or periodic in-person visits to the specialist centre [9]. The costs of implementing CMH shared-care were not reported for any of the programs (0/8).

Various implementation strategies were used to support shared-care delivery as intended. Slater et al. [24] created a governing network to provide oversight for their model, inform care delivery, oversee quality improvement initiatives, and support research and education at specialist centres and community hospitals. Additional strategies included patient evaluation tools [25], joint electronic medical records [23,24], daily patient care discussions [18], dedicated treatment areas for immune-compromised patients [24], and patient education about community hospital and specialist centre responsibilities [9,24,25,26]. Challenges included a lack of available resources to provide the required care in the community [18], long wait times associated with patient transfer between centres [18], and the lack of available beds in specialized centres when hospitalization was necessary [18]. Possible solutions were proposed, such as the establishment of consultation services at both sites, including transfusion medicine and blood bank support, infectious diseases, and critical care [18,21], and ‘backup’ patient transfer service availability for community hospitals [22].

### 3.5. Maintenance

The maintenance of a program considers the measurement of the sustainability of an intervention [10]. Maintenance indicators (outcomes > 6 months post-intervention, indicators of program maintenance, or measures of cost of maintenance) were not reported for any of the programs (0/8). 

## 4. Discussion

In this integrative review, the RE-AIM framework was used to evaluate what is known about the implementation of CMH shared-care. Eight programs, described across ten articles, were included in this review. Indicators of reach were reported for 65% of the programs, and emphasized standardized referrals and some eligibility criteria; however, patient participation rate and methods for identifying eligible patients were lacking. Effectiveness indicators were reported for 28% of programs, and suggested favourable survival outcomes within a shared-care model; however, health system impact and quality of life studies were lacking. Indicators of adoption and implementation were reported for 56% and 42% of programs, respectively, and emphasized program components of a multidisciplinary team, relationship-building, pre-planned protocols for managing patients at each site, infrastructure support, and various communication strategies such as shared electronic health systems. Measures of cost and maintenance were not reported. 

Because the methods for identifying patients were limited to specialist centre selection, there is a lack of understanding of who might benefit from shared-care, from the community hospital perspective. Known clinician-related referral barriers include knowledge gaps in relation to eligibility for various therapies, as well as a lack of established referral criteria [27,28]. Subjective interpretation of fitness for therapy, including age, comorbidities, and functional status, may impact patient referral. Some patients may not be offered evidence-based therapies if methods to identify patients have not been well established. As identified by our review, establishing referral criteria and networking between hospital sites may be an effective strategy for patient identification. 

The rate of patient participation was also poorly reported, limiting our understanding of the reasons that eligible patients may or may not receive shared-care. Known patient-related referral barriers to a specialist centre include hesitancy for specific treatment, travel burdens, financial challenges, or lack of caregiver support [5,27,28]. There may be additional factors that influence patient decision-making related to the site of care. Further research to better understand participation rate and strategies to target patient-related referral barriers may help with program recruitment and retention among eligible patients. 

Despite common patient eligibility criteria for CMH shared-care across programs (e.g., treatment subtypes), important details were lacking, providing little information about the ideal patient for shared-care. For example, the presence of opportunistic infections, requiring consultation with specialized infectious disease experts, may be important to consider when determining eligibility for shared-care. Criteria for treating CMH patients in the outpatient setting have been established to minimize the risk of treatment-related mortality, suggesting that a similar model can be instituted for shared-care. For example, Mabrey et al. [29] defined eligibility for outpatient management of AML patients as having adequate cardiac function, normal chest imaging, low disease burden, residing within 30 min of the hospital, and dedicated caregiver support. Research to determine the most appropriate eligibility criteria for shared-care will be important to optimize the safety and potential benefits of these programs. 

CMH patients treated at specialist centres have improved survival outcomes compared to patients treated in community hospitals [30,31]. This may be attributed to increased resources, staff expertise, and availability of evidence-based therapies at the specialist centre [32,33]. In our review, those who studied the effect of CMH shared-care compared to patients treated entirely at a specialized centre or community hospital, found improved or comparable survival outcomes, suggesting this model may be an effective strategy for improving access to care. Future studies should have larger sample sizes and reduced risk of selection bias, to verify these findings. Measurement of quality of life would provide a better sense of the impact of CMH shared-care on patients’ perceptions of physical, emotional, and social health. Travel time can be burdensome for patients; however, patients prefer quality specialist care, and they are more likely to choose therapy at specialized centres if follow-up and supportive care are shared locally with community hospitals [34,35]. Further research from the patient’s perspective would help inform an understanding of whether shared-care programs effectively improve quality of life. 

Across programs, the characteristics of the specialist centre were well-described; however, few programs reported on the characteristics of the community hospitals, limiting our understanding of the infrastructure that would support a shared-care model. Community hospitals and specialist centres have varying levels of service provision [36], and it is unclear from the literature what infrastructure is essential to consider. Ontario Health [36] has developed organizational requirements for providing acute leukemia care, which include the availability of private rooms for isolation, and outpatient assessment areas that can reasonably protect patients from transmission of infectious agents. Inclusion and exclusion criteria such as this would help decision-makers assess their organization’s readiness and advocate for the necessary resources to participate in shared-care. 

Our review emphasized the challenge of maintaining staff expertise in the community setting, where CMH patient volumes might be low. Staff and program leadership are responsible for ensuring clinicians demonstrate competency in prescribing, monitoring, and managing the complications of various therapies. From our review, facilitators included a trained and multidisciplinary team of providers, regular educational opportunities, and access to timely expert clinical advice. Strategies, such as physician sub-specialization, could be explored, as this may facilitate confidence in clinical management for CMH patients [37]. Additionally, investigating alternate primary provider roles, such as nurse practitioners, may be valuable in providing continuity, increasing the level of staff expertise [38].

Although implementation fidelity was not well reported, the literature highlighted important elements for incorporating shared-care programs into the health system. Resources such as consultation services, patient education, joint electronic health records, and virtual care were identified as important program components. For providing acute leukemia services, Ontario Health [36] has identified the need for 24 h access to irradiated blood products, medications needed for CMH patients (e.g., all-trans retinoic acid), and diagnostic services such as bronchoscopy. While this provides important considerations for CMH shared-care program planning, the literature lacks a complete evaluation of these components. Additionally, virtual care increases access to care in oncology, and improves patient satisfaction and health outcomes [39], but how virtual care can be used in the context of CMH shared-care is less known. Further research could explore the organizational resources required for shared-care, including how virtual care can be used effectively in this patient population. 

None of the articles included in our review reported on aspects of CMH shared-care maintenance, limiting our understanding of program sustainability and whether shared-care can be successfully integrated into health systems. Muir et al. [22] identified that certain funding models that split finances between the specialist centre and community hospital could be a potential barrier to implementation; allocated funds into the community may compromise the ability of specialist centres to maintain specialized staff and resources. However, Muir et al. [22] also indicated that shared-care reduced specialist centre volumes and workload, enabling clinicians to devote time to caring for more complex patients. Economic evaluation studies may help to understand the financial impacts that would result from the implementation of shared-care, and inform an understanding of the resources required for long-term program delivery. For example, Cohen et al. [7] reported a reduction in health resource utilization with the introduction of a shared-care model for children with chronic conditions.

### Limitations and Strengths

There are limitations to this review. First, the search was conducted using a variety of synonymous terms for shared-care, such as co-management or hub-and-spoke; however, this terminology is not consistent throughout the literature, thus it is possible some articles reporting a CMH shared-care program were missed. Second, conference abstracts and the grey literature were excluded from the eligibility criteria, limiting our understanding of CMH shared-care’s full scope. Third, the lack of standard reporting of implementation in the CMH shared-care literature (e.g., according to StaRI [40] guidelines), decreases the ability to compare programs effectively. A key strength was using the RE-AIM data extraction tool, which ensured a comprehensive understanding of the reporting of indicators. All searches were conducted at the same time and there were no start date limitations. 

## 5. Conclusions

As the CMH patient population grows and ages, the challenges of travel burdens persist, and specialist centres face ongoing capacity pressures, it is critical to explore shared-care models as a potential solution. In this integrative review, the RE-AIM framework provided a guide for evaluating what is known about CMH shared-care programs. Favourable survival outcomes within a shared-care model were emphasized, and important program components included a multidisciplinary team, relationship-building, and shared communication strategies. Further research is warranted on patient eligibility, rationale for participation, health system and quality of life outcomes, maintaining staff expertise, infrastructure support, virtual care, and measures of cost and sustainability. 

## Figures and Tables

**Table 1:** Definitions of RE-AIM Dimensions.

RE-AIM Dimension	Definition	Importance
Reach	Assesses the number, proportion, and representativeness of patients willing to participate in CMH shared-care [10]	Helps to determine who may be suitable for CMH shared-care, and provides information on the acceptability of the intervention from the patient’s perspective [15]
Effectiveness	Evaluates the impact of CMH shared-care on important outcomes (measures/results, quality of life, unintended consequences) [10]	Evaluates whether CMH shared-care outcomes were changed (positively or negatively) [15]
Adoption	Assesses the number, proportion, and representativeness of settings and staff who deliver CMH shared-care [10]	Helps to determine which settings are most suitable for CMH shared-care, and provides information on the approach to identify and engage staff for program delivery [15]
Implementation	Refers to intervention duration and frequency, the extent CMH shared-care was delivered as intended, measures of implementation cost [10]	Provides insight into the resources needed compared to the resources available, as well as the feasibility of delivering all components of CMH shared-care [15]
Maintenance	Evaluates the degree to which CMH shared-care is continued as part of organizational practices and policies [10]	Provides insight into whether the intervention can be integrated into health systems [15]

(CMH = complex malignant hematology).

**Table 2:** Example of the Search Strategy used in Medline.

Population	Complex malignant hematology terms leukemia (meSH) OR leuk?emi* OR lymphoma (meSH) OR lymphoma* OR myelodysplastic syndromes (meSH) OR myelodysplas* OR myelo-dysplas* OR myeloproliferative disorders (meSH) OR myeloprolif* OR myelo-prolifer* OR myelodysplastic-myeloproliferative diseases (meSH) OR multiple myeloma (meSH) OR myeloma OR hematologic neoplasms (meSH) OR hematopoietic stem cell transplantation (meSH) OR transplantation, autologous (meSH) OR (autologous AND transplant*) OR transplantation, homologous (meSH) OR ((homologous OR allogeneic) AND transplant*) OR receptors, chimeric antigen (meSH) OR immunotherapy, adoptive (meSH) OR (chimeric AND antigen AND receptor AND therapy) OR induction chemotherapy (meSH) OR consolidation chemotherapy (meSH) OR maintenance chemotherapy (meSH)
AND Intervention	Shared-care terms ((share? OR sharing?) AND care) OR co-management OR (care AND coordination) OR hospital shared services (meSH) OR (hospital AND (shar? OR sharing?) AND service*) OR delivery of health care, integrated (meSH) OR (integrated adj3 healthcare) OR community networks (meSH) OR (community AND network*) OR (community and partner*) OR cooperative behavior (meSH) OR (hub AND spoke) OR (hospitals, community (meSH) OR hospitals, general (meSH) OR hospitals, low-volume (meSH) OR hospitals, rural (meSH) OR hospitals, centers (meSH)) AND (hospitals, teaching (meSH) OR hospitals, urban (meSH) OR tertiary care centers (meSH)) OR (hospitals, high-volume (meSH) OR cancer care facilities (meSH))

**Table 3:** Characteristics of Included Programs.

Author (Year)	Study Location	Aim	Design	Participants	Description of Patient-Sharing Model
Cheung et al. (2021) [25]	Canada	To describe the shared-care model between specialized cancer centre and local hospital for patients with MPN	Discussion paper	Adult; MPN	Treatment decisions and some therapy at specialized centre; supportive care at local hospital (e.g., count checks, transfusion support)
Goradia et al. (2023) [23]	New York, USA	To describe a shared cancer care delivery model for patients with myeloid malignancies between academic leukemia centre and general community oncologists	Case series of patients	Adult, MDS and acute leukemia	Initial patient visit in person or via telehealth at specialist centre, and subsequent care delivered at community hospital.
Hershenfeld et al. (2017) [9]	Canada	To review the impact of shared-care model with specialized cancer centre and local hospitals with an emphasis on travel time and distance saved	Retrospective cohort study	Adult, AML	Patients receive post-consolidation supportive therapy at local hospital, while consolidation chemotherapy itself is administered at specialist centre
Jillella and Kota (2018) [20] Jillella et al. (2020) [19] Jillella et al. (2021) [18]	Georgia, USA	To describe a strategy of co-management by community oncologists and APL experts and implementation of standardized treatment algorithm to reduce early deaths in APL	Discussion paper (n = 2); Prospective cohort study (n = 1)	Adult, APL	Co-management between 4 large leukemia treatment centres and 15 local community hospitals, with a focus on physician education and support (via phone/email) for managing newly diagnosed APL in the community.
Law et al. (2021) [21]	California, USA	A pre- and post-implementation evaluation comparing patients from 2013/2014 (managed by community-based hematologists) to 2016/2017 after shared-care was implemented.	Retrospective cohort study	Adult, AML	Patient-sharing between regional leukemia centre and local centre; with focus on referrals, identifying and shifting less-intensive therapies to community
Lim et al. (2022) [26]	Singapore	To describe the development and implementation of a hub-and-spoke model of cross border patient-sharing collaboration for CAR-T therapy (Singapore as hub and East Asian countries as spoke centres)	Discussion paper	Adult, lymphoma	Patients receive standard therapy in home country, CAR-T in hub treatment centre, and then back to spoke country for post therapy monitoring
Muir et al. (1992) [22]	England, UK	To describe the survival of patients with acute lymphoblastic leukemia treated in shared-care model with regional hospitals	Nested case-control study	Pediatric, ALL	Children are referred to regional specialist centre for initial diagnosis and treatment; management of continuing treatment is carried out at regional hospital
Slater et al. (2022) [24]	Australia	To examine the role of regional case managers with patient sharing in tertiary centre and shared-care sites	Qualitative, phenomenological study	Pediatric, malignant hematology	Tertiary children’s hospital and network of 10 local shared-care sites. Shared-care sites provide low-risk chemotherapy and supportive care after diagnosis, care planning, and some treatment is performed at tertiary centre.

(CAR-T = chimeric antigen receptor therapy; MPN = myeloproliferative neoplasm; MDS = myelodysplastic syndrome; AML = acute myeloid leukemia; APL = acute promyelocytic leukemia; ALL = acute lymphoblastic leukemia).

**Table 4:** Proportion of CMH Shared-Care Programs Reporting RE-AIM Dimensions and Indicators.

RE-AIM Dimensions and Indicators	Frequency	Proportion
**Reach: The number, proportion, and representativeness of patients willing to participate in CMH shared-care**		
Method to identify patients	6/8	
Inclusion criteria	8/8	
Exclusion criteria	2/8	
Sample size and participation rate	5/8	
Characteristics of both participation and non-participation	4/8	
Average of overall reach dimension	26/40	65%
**Effectiveness: The impact of CMH shared-care on important outcomes**		
Measures/results	6/8	
Intent-to-treat analysis utilized	0/8	
Quality of life outcomes	2/8	
Percent attrition	1/8	
Average of overall effectiveness dimension	9/32	28%
**Adoption: The number, proportion, and representativeness of settings, and staff who deliver CMH shared-care**		
Description of intervention location	8/8	
Description of staff who delivered intervention	7/8	
Method to identify staff who delivered CMH shared-care	3/8	
Level of staff expertise	7/8	
Inclusion/exclusion criteria of shared-care setting	2/8	
Adoption rate	0/8	
Average of overall adoption dimension	27/48	56%
**Implementation: Fidelity to various elements of CMH shared-care**		
Intervention duration and frequency	2/8	
Extent shared-care delivered as intended	8/8	
Measures of cost of implementation	0/8	
Average of overall implementation dimension	10/24	42%
**Maintenance: Extent to which CMH shared-care is maintained after intervention**		
Assessed outcomes >6 months post-intervention	0/8	
Current status of program	0/8	
Measures of cost of maintenance	0/8	
Average of overall maintenance dimension	0/24	0%

**Table 5:** Programs Reporting Effectiveness.

Author (Year)	Patients	Results
Hershenfeld et al. (2017) [9]	n = 417; 73 patients received shared-care vs. 344 patients treated only at specialist centre	No significant difference in survival between 2 groups (90 d survival = 95.9% vs. 95.3%). No significantly increased hazard of death found for shared-care group.
Jillella et al. (2021) [18]	n = 118 (73 shared-care patients vs. 45 treated only at specialist centre)	No difference in induction mortality between 2 groups of patients (8.2% in shared-care vs. 8.8% at specialist centre alone) and no difference in 1-year survival. Overall 1-year survival rate for whole group was 87.3% (superior in comparison to 70.7% reported in SEER data)
Law et al. (2021) [21]	n = 249 (135 shared-care and 114 treated at community hospital alone) in 2016/2017 vs. n = 278 treated at community hospital alone in 2013/2014	More patients received induction therapy (intensive and less-intensive inductions) with implementation of regionalization (65.2% vs. 49%). Observed reductions in 60 d (HR = 0.67) and 180 d mortality (HR = 0.64) in comparison to time period prior to shared-care implementation.
Muir et al. (1992) [22]	n = 146 (49 shared-care vs. 97 treated at specialist centre alone)	When age-matched with comparison group, the 49 patients included in shared-care model had survival rates comparable to those treated entirely at specialist centre *.

* The paper illustrated a survival curve; however, exact percentages were not included.

## Data Availability

No new data were created or analyzed in this study. Data sharing is not applicable to this article.

## Supplementary Materials

The following supporting information can be downloaded at: https://www.mdpi.com/article/10.3390/curroncol31090406/s1, S1. Search Strategies. S2. Reporting of RE-AIM Indicators.
