# Supplementary material for: Shared-Care in Complex Malignant Hematology: An Integrative Review Using the RE-AIM Evaluation Framework
Source: Curr Oncol. 2024 Sep 14;31(9):5484–97. doi: 10.3390/curroncol31090406 (PMC11431418; doi:10.3390/curroncol31090406)
Supplement: Supplementary file 1 [file curroncol-31-00406-s001.zip › curroncol-3167425-supplementary.pdf]

S1: Search Strategies

MEDLINE SEARCH STRATEGY 16 January 2024

NO	SEARCH TERMS	RESULTS
1	exp Leukemia/	256,457
2	Leuk?emi*.mp,kw	369,976
3	1 or 2	371,777
4	exp Lymphoma/	189,287
5	Lymphoma*.mp,kw	285,585
6	4 or 5	307,922
7	exp Myelodysplastic syndromes/	24,070
8	(myelodysplas* or myelo-dysplas*).mp,kw	27,265
9	7 or 8	33,369
10	exp Myeloproliferative Disorders/	51,683
11	(myeloprolif* or myelo-prolifer*).mp,kw	16,577
12	10 or 11	57,219
13	exp Myelodysplastic-Myeloproliferative Diseases/	2665
14	exp Multiple Myeloma/	48,494
15	Myeloma.mp,kw	72,150
16	14 or 15	72,675
17	exp Hematologic Neoplasms/	25,623
18	(h?ematolog* and neoplasm).mp,kw	14,208
19	17 or 18	37,794
20	exp Hematopoietic Stem Cell Transplantation/	58,246
21	exp Transplantation, Autologous/	54,104
22	(autologous and transplant*).mp,kw	86,226
23	21 or 22	86,226
24	exp Transplantation, Homologous/	91,309
25	(homologous or allogeneic) and transplant*)).mp,kw	122,509
26	24 or 25	127,007
27	exp Receptors, Chimeric Antigen/	4749
28	exp Immunotherapy, Adoptive/	14,300
29	chimeric antigen receptor therapy.mp	77
30	27 or 28 or 29	15,689
31	exp Induction Chemotherapy/	3911
32	exp Consolidation Chemotherapy/	754
33	exp Maintenance Chemotherapy/	2140
34	3 or 6 or 9 or 12 or 13 or 16 or 19 or 20 or 23 or 26 or 30 or 31 or 32 or 33	**889,910**
35	((share? Or sharing?) and care).mp,kw	68,733
36	Co-management.mp,kw	832
37	(care and coordination).mp,kw	20,695
38	Hospital Shared Services/	2159
39	(hospital and (shar? Or sharing?) and service*).mp,kw	5014
40	38 or 39	6991
41	exp “Delivery of Health Care, Integrated”/	14,558
42	(integrated adj3 healthcare).mp,kw	2536
43	41 or 42	16,538
44	Community Networks/	7198
45	(community and network*).mp,kw	43,867
46	44 or 45	43,867

47	(community and partner*).mp,kw	32,900
48	Hospitals, community/or hospitals, general/or hospitals, low-volume/or hospitals,rural or hospitals/satellite	33,775
49	Hospitals, high-volume/or cancer care facilities/or exp hospitals, teaching/or exp hospitals,urban/or tertiary care centers/	92,978
50	48 and 49	4001
51	Cooperative Behavior/	46,180
52	(hub and spoke).mp,kw	841
53	35 or 36 or 37 or 40 or 43 or 46 or 47 or 48 or 50 or 51 or 52	**247,287**
54	34 and 53	**1215**

EMBASE SEARCH STRATEGY 16 January 2024

NO	SEARCH TERMS	RESULTS
1	exp leukemia/	374,243
2	exp myelodysplastic syndrome/	53,095
3	exp multiple myeloma/	99,994
4	exp myeloproliferative neoplasm/or exp myeloproliferative disorder/	508,869
5	leukemia*.mp	550,191
6	lymphom*.mp	408,784
7	(myelodysplas* or myelo-dysplas*).mp	60,918
8	(myeloprolif* or myelo-prolif*.mp	30,422
9	myeloma.mp	130,028
10	exp hematopoietic stem cell transplantation/	90,370
11	exp allogeneic stem cell transplantation/	54,163
12	exp autologous stem cell transplantation/	33,616
13	exp chimeric antigen receptor T-cell immunotherapy/	10,921
14	Car-t therapy.mp	3153
15	exp induction chemotherapy/	18,868
16	exp consolidation chemotherapy/	4850
17	exp maintenance chemotherapy/	3785
18	co-management.mp	1480
19	(care and coordination).mp	34,440
20	(hospital and shared and service*).mp	5655
21	(integrated and health and care).mp	77,883
22	(community and network*).mp	51,437
23	(community and partner*).mp	43,564
24	exp community care/	133,198
25	(hub and spoke).mp	1636
26	exp community hospital/	21,219
27	exp teaching hospital/	227,538
28	26 and 27	2358
29	exp lymphoma/	332,733
30	1 or 2 or 3 or 4 or 5 or 6 or 7 or 8 or 9 or 10 or 11 or 12 or 13 or 14 or 15 or 16 or 17 or 29	**110,4861**
31	Shared-care.mp	2780
32	(shared and care).mp	57,868
33	18 or 19 or 20 or 21 or 22 or 23 or 24 or 25 or 26 or 28 or 31 or 32	**385,137**
34	30 and 33	**3021**

COCHRANE SEARCH STRATEGY (Cochrane Central Register of Controlled Trials and Cochrane Database of Systematic Reviews) 16 January 2024

NO	SEARCH TERMS	RESULTS
1	leukemia.mp [mp=ti, ot, ab, fx, sh, hw, kw, tx, ct]	15,751
2	lymphoma.mp [mp=ti, ot, ab, fx, sh, hw, kw, tx, ct]	13,755
3	myeloma.mp [mp=ti, ot, ab, fx, sh, hw, kw, tx, ct]	6691
4	(myelodysplas* or myelo-dysplas*).mp	2881
5	(myeloprolif* or myelo-prolif*).mp	623
6	(hematology* and neoplasm).mp	1627
7	(autologous and transplant*).mp	7908
8	((allogeneic or homologous) and transplant*).mp	5863
9	(chimeric and antigen and receptors).mp	57
10	(CAR-T and therapy).mp	286
11	1 or 2 or 3 or 4 or 5 or 6 or 7 or 8 or 9 or 10	**40,958**
12	((share or shared) and (car* or care*)).mp	8038
13	co-management.mp	65
14	(care and coordination).mp	2334
15	(hospital and shar* and service*).mp	2175
16	(integrated and healthcare).mp	2001
17	(community and network*).mp	3543
18	(community and partner*).mp	3939
19	(hub and spoke).mp	69
20	(community and hospital).mp	11,223
22	(teaching or urban or tertiary) and hospital).mp	18,652
22	20 and 21	1957
23	12 or 13 or 14 or 15 or 16 or 17 or 18 or 19 or 20 or 22	**26,721**
24	11 and 23	**351**

CINAHL SEARCH STRATEGY 16 January 2024

NO	SEARCH TERMS	RESULTS
1	(MH “Leukemia+”)	26,156
2	(MH “Lymphoma+”)	31,917
3	(MH “Multiple Myeloma+”)	7913
4	(MH “Myeloproliferative Disorders+”)	2129
5	(MH “Myelodysplastic Syndromes”)	2542
6	(MH “Hematologic Neoplasms+”)	4444
7	(MH “Hematopoietic Stem Cell Transplantation”)	10,370
8	(MH “Bone Marrow Transplantation+”)	4910
9	(MH “Bone Marrow Transplantation, Allogeneic”)	263
10	(MH “Bone Marrow Transplantation, Autologous”)	263
11	TI (chimeric AND antigen AND receptor AND therapy) OR AB (chimeric AND antigen AND receptor AND therapy)	1339
12	TI (CAR-T AND therapy) OR AB (CAR_T AND therapy)	1430
13	(MH “Induction Chemotherapy”)	114
14	(MH “Consolidation Chemotherapy”)	54
15	TI (maintenance AND chemotherapy) RO AB (maintenance AND chemotherapy)	1979
16	TI (leuk?emi* OR lymphoma* OR myelom*) OR AB (leuk?emi* OR lymphoma* OR myelom*)	48,171
17	TI (hematology* AND neoplasm*) OR AB (hematology* AND neoplasm*)	685

18	S1 or S2 or S3 or S4 or S5 or S6 or S7 or S8 or S9 or S10 or S11 or S12 or S13 or S14 or S15 or S16 or S17	**94,023**
19	(MH "Shared Services, Health Care")	671
20	TI (shar* AND care) OR AB (shar* AND care)	36,060
21	TI co-management OR AB co-management	359
22	TI (care AND coordination) OR AB (care AND coordination)	11,630
23	(MH "Health Care Delivery, Integrated")	15,359
24	(MH "Community Networks")	2672
25	TI (community AND partner*) OR AB (community AND partner*)	20,269
26	(MH "Cooperative Behavior")	9052
27	TI (hub AND spoke) OR AB (hub AND spoke)	366
28	TI shared-care OR AB shared-care	1032
29	(MH "Regional Centers")	257
30	(MH "Hospitals, Community")	5728
31	(MH "Hospitals, Rural")	3267
32	(MH "Academic Medical Centers")	82,692
33	(MH "Cancer Care Facilities")	6664
34	S19 or S20 or S21 or S22 or S23 or S24 or S25 or S26 or S27 or S28 or S29 or S30 or S31 or S32 or S33	**184,922**
35	S18 AND S34	**1649**

SCOPUS SEARCH STRATEGY 16 January 2024

((TITLE-ABS-KEY (car-t AND therapy OR chimeric AND antigen AND receptor AND therapy)) OR (TITLE-ABS-KEY (autologous AND stem AND cell AND transplant OR allogeneic AND stem AND cell AND transplant OR hematopoietic AND stem AND cell AND transplant)) OR (TITLE-ABS-KEY (myelodysplastic AND syndromes OR myeloproliferative AND neoplasm)) OR (TITLE-ABS-KEY (multiple AND myeloma)) OR (TITLE-ABS-KEY (lymphoma)) OR (TITLE-ABS-KEY (leukemia)))

AND ((TITLE-ABS-KEY (shared-care OR shared AND care)) OR (TITLE-ABS-KEY (co-management OR care AND coordination OR hospital AND shared AND services OR integrated AND health AND care)) OR (TITLE-ABS-KEY (community AND networks OR community AND partner OR hub AND spoke)) OR (TITLE-ABS-KEY (community AND networks OR community AND partner OR hub AND spoke)) OR (TITLE-ABS-KEY (community AND hospital OR regional AND hospital)) OR ((TITLE-ABS-KEY (teaching AND hospital OR tertiary AND hospital OR academic AND hospital)) AND (TITLE-ABS-KEY (community AND hospital OR regional AND hospital

S2: Reporting of RE-AIM Indicators

Cheung et al. (2021) [25]			
RE-AIM Indicators	Reported? (Yes, No)	Data	Facilitators/Barriers
**Reach: The absolute number, proportion, and representatives of individuals who are willing to participate in CMH shared-care**			
Method to identify patients	Yes	Patients identified at specialist centre by external referrals	Facilitator: standardized referral form to ensure efficiency and accuracy in referral prioritizations
Inclusion criteria	Yes	Target population identified by MPN referral criteria	Facilitator: standardized referral form to ensure accuracy in referral
Exclusion criteria	No		
Sample size and participation rate	No		
Characteristics of both participation and non-participation	No		
**Effectiveness: The impact of CMH shared-care on important outcomes**			
Measures/results (at shortest assessment)	No		
Intent-to-treat analysis utilized	No		
Quality of life outcomes	No		
Percent attrition	No		
**Adoption: The absolute number, proportion, and representativeness of settings, and people who deliver CMH shared-care who are willing to initiate the program**			
Description of intervention location	Yes	Described specialist centre characteristics (e.g., enhanced diagnostic abilities), and community hospital role	
Description of staff who delivered intervention	Yes	CNS identified as lead, and role described	Facilitator: Given broad scope of practice, able to act as a resource to nurses, patients, and caregivers and participate in entire patient care trajectory
Method to identify staff who delivered intervention (target delivery agent)	No		
Level of staff expertise	Yes	CNS is a master's prepared nurse with expertise in MPNs	
Inclusion/exclusion criteria of delivery agent/setting	No		
Adoption rate	No		
**Implementation: The intervention agents' fidelity to the various elements of an intervention protocol (including implementation strategy)**			

Intervention duration and frequency	No		
Extent shared-care delivered as intended	Yes	Triage new referrals, see patients in consultation<br>Communication of the expectations for shared-care with patients.<br>Communication of responsibilities with shared-care partner sites.<br>Referring to palliative care or psychosocial support<br>Patient education re: Disease<br>Care coordination<br>Medication management, symptom management	Facilitators: having clinic capacity, dedicated interdisciplinary team members, appropriate evaluation tools (MPN symptom survey), clear communication of expectations for shared-care with patients and community partners
Measures of cost of implementation	No		
**Maintenance: The extent to which program is institutionalized at some duration after intervention (includes reasons for maintenance, discontinuation, or adaptation)**			
Assessed outcomes >6 months post-intervention	No		
Current status of program	No		
Measures of cost of maintenance	No		
**Goradia et al. (2023) [23]**			
**RE-AIM Indicators**	Reported? (Yes, No)	Data	Facilitators/Barriers
**Reach: The absolute number, proportion, and representatives of individuals who are willing to participate in CMH shared-care**			
Method to identify patients	Yes	Referred for 2nd opinion related to new diagnosis or follow-up of MDS/AML	
Inclusion criteria	Yes	Patients were either elderly and/or poor performance status	
Exclusion criteria	No		
Sample size and participation rate	Yes	N = 12. Participation rate—NR	
Characteristics of both participation and non-participation	Yes	Age range 59–88; 67% had ECOG 2 or higher. Did not describe the patients who were not included in co-management model	
**Effectiveness: The impact of CMH shared-care on important outcomes**			
Measures/results (at shortest assessment)	Yes	No statistical analysis performed, but descriptive statistics; median # of hospitalizations during	

		treatment period was 1 (range 1–3). Median time patients remained on treatment was 357 days (range 154–557)	
Intent-to-treat analysis utilized	No		
Quality of life outcomes	No		
Percent attrition	No		
**Adoption: The absolute number, proportion, and representativeness of settings, and people who deliver CMH shared-care who are willing to initiate the program**			
Description of intervention location	Yes	Described academic, NCI cancer centre and community hospitals	
Description of staff who delivered intervention	Yes	Description of specialist MD and primary oncologist	
Method to identify staff who delivered intervention (target delivery agent)	No		
Level of staff expertise	Yes	Partial description of specialist MD, with subspecialty in leukemia	Facilitator: Bi-weekly virtual conferences to discuss challenging cases (both academic and community physicians)
Inclusion/exclusion criteria of delivery agent/setting	Yes	Community oncology practices were an established partnership with academic centre	
Adoption rate	No		
**Implementation: The intervention agents' fidelity to the various elements of an intervention protocol (including implementation strategy)**			
Intervention duration and frequency	Yes	# of shared-care visits (median of 3.5 in-person and 3 telehealth) described during intervention	
Extent shared-care delivered as intended	Yes	Admitted to specialist centre for leukoreduction, otherwise received all care at local centre with telehealth visits q8-10 weeks<br>In-person visits PRN and as per patient preference<br>Treatment plans documented in shared EPR<br>Community oncologists given 24/7 access to communicate with specialist physician	Facilitators: shared electronic health record to document labs, notes, imaging etc. Use of telehealth to deliver shared-care intervention. Many approved drugs for elderly are oral agents, making it easier to manage patients via telehealth.<br><br>Barriers: inability to examine patient with telehealth visit
Measures of cost of implementation	No		
**Maintenance: The extent to which program is institutionalized at some duration after intervention (includes reasons for maintenance, discontinuation, or adaptation)**			

Assessed outcomes > 6 months post-intervention	No	Implementation initiated March 2020 in light of COVID-19 pandemic.	
Current status of program	No		
Measures of cost of maintenance	No		
**Hershenfeld et al. (2017) [9]**			
**RE-AIM Indicators**	Reported? (Yes, No)	Data	Facilitators/Barriers
**Reach: The absolute number, proportion, and representatives of individuals who are willing to participate in CMH shared-care**			
Method to identify patients	Yes	Patients identified by nurse coordinators, NPs, physicians based on geographic location.	
Inclusion criteria	Yes	All patients had AML or APL diagnosis and were receiving post-consolidation care in CR1	
Exclusion criteria	Yes	Patients were encouraged to participate, but not mandatory.	
Sample size and participation rate	Yes	73/344 patients participated in shared-care model. No comment on how many were eligible, but did not participate; participation rate= NR	
Characteristics of both participation and non-participation	Yes	No significant differences were found between demographic and cytogenetic characteristics between 2 groups	
**Effectiveness: The impact of CMH shared-care on important outcomes**			
Measures/results (at shortest assessment)	Yes	Survival—no significant difference	
Intent-to-treat analysis utilized	No		
Quality of life outcomes	Yes	Travel time—reduced for shared-care patients<br><br>Patients saved an estimated 9.7 h and 882 km of travel time and distance during 1 consolidation cycle	
Percent attrition	No		
**Adoption: The absolute number, proportion, and representativeness of settings, and people who deliver CMH shared-care who are willing to initiate the program**			
Description of intervention location	Yes	Description of quaternary cancer centre and 14 regional hospitals	

Description of staff who delivered intervention	Yes	Staff at regional hospitals included medical oncologists, hematologists, NPs	
Method to identify staff who delivered intervention (target delivery agent)	Yes	Partnerships with regional hospitals were establishing through communication and training	
Level of staff expertise	Yes	Description of staff with experience in the treatment of cytopenias and febrile neutropenia	Facilitators: Annual education days were held at both partner sites and quaternary centre to review protocols and support multidisciplinary team; on-call rosters were published for timely physician-physician communication between sites; guidelines were made available for care items such as transfusion thresholds etc.
Inclusion/exclusion criteria of delivery agent/setting	No		
Adoption rate	No		
**Implementation: The intervention agents' fidelity to the various elements of an intervention protocol (including implementation strategy)**			
Intervention duration and frequency	No		
Extent shared-care delivered as intended	Yes	Shared-care patients underwent 137 cycles of consolidation, with post-consolidation care at 14 regional hospitals. Each site treated median of 2 patients<br><br>Patients were given a letter with details outlining their therapy protocol to bring with them to local centre.<br><br>Local sites were advised to contact specialist centre when patient was admitted unexpectedly, patients were treated for FN at local centre and only transferred if complications warranted.	Facilitator: guidelines for frequency of blood count checks, transfusion thresholds, CVC maintenance, and symptom management including febrile neutropenia
Measures of cost of implementation	No		
**Maintenance: The extent to which program is institutionalized at some duration after intervention (includes reasons for maintenance, discontinuation, or adaptation)**			
Assessed outcomes >6 months post-intervention	No		

Current status of program	No		
Measures of cost of maintenance	No		
**Jillella and Kota (2018); Jillella et al. 2020); Jillella et al. (2021) [18–20]**			
**RE-AIM Indicators**	Reported? (Yes, No)	Data	Facilitators/Barriers
**Reach: The absolute number, proportion, and representatives of individuals who are willing to participate in CMH shared-care**			
Method to identify patients	Yes	Patients who presented to community hospitals were enrolled if an APL expert was contacted at the time of diagnosis	
Inclusion criteria	Yes	Patients with confirmed diagnosis of APL and receiving standard therapy. Patients were consented to collect treatment data	
Exclusion criteria	No		
Sample size and participation rate	Yes	73/118 were managed in the community; 45 patients treated only at specialist centre. Participation rate: NR;	
Characteristics of both participation and non-participation	Yes	Median age of all 118 patients—52.5 years.	
**Effectiveness: The impact of CMH shared-care on important outcomes**			
Measures/results (at shortest assessment)	Yes	No difference in induction mortality between 2 groups of patients (8.2% vs. 8.8%) and no difference in 1-year survival.<br><br>Overall 1-year survival rate for whole group was 87.3% (superior in comparison to 70.7% reported in SEER data)	
Intent-to-treat analysis utilized	No		
Quality of life outcomes	No		
Percent attrition	No		
**Adoption: The absolute number, proportion, and representativeness of settings, and people who deliver CMH shared-care who are willing to initiate the program**			
Description of intervention location	Yes	Description of tertiary leukemia centre described, and community hospitals	

Description of staff who delivered intervention	Yes	Defined as APL experts and community oncologists	
Method to identify staff who delivered intervention (target delivery agent)	Yes	Visited centres<br><br>Accrual increased as the trial went on—as many referring physicians did not call the specialist physicians initially. Initially, community oncologists did not think early deaths was a problem in APL	Facilitator: aggressive outreach effort was made to visit community hospital and provide education—physically visiting the community helped to build a relationship; program awareness contributed to higher accrual; in time—support for the program became stronger; participating in state and regional meetings strengthened collaboration
Level of staff expertise	Yes	Training was provided	Facilitator: developing a simple 2-page treatment algorithm highlighting methods to prevent complications; having centralized expertise and opportunity for community hospitals to seek early advice (direct and easy access—cell phone numbers available 24/7); clinicians want to treat to maintain leukemia treatment skills given the rarity of disease<br><br>Barrier: treating leukemia requires familiarity and trained and dedicated nursing staff/oncology pharmacists—leukemia accounts for <3% of all cancers
Inclusion/exclusion criteria of delivery agent/setting	No		
Adoption rate	No	Did comment on the expansion of the trial to other regions d/t growing awareness	
**Implementation: The intervention agents' fidelity to the various elements of an intervention protocol (including implementation strategy)**			
Intervention duration and frequency	Yes	Awareness/education occurred over 6-month period	
Extent shared-care delivered as intended	Yes	2-page treatment algorithm implemented (guidelines for starting ATRA, steroids etc)<br><br>Education program to increase awareness of early deaths associated with APL implemented<br><br>Communication with both physicians re: patient progress daily x2 weeks, then q2-3d until discharge	Facilitators: aggressive outreach effort was made by visiting both tertiary and community hospitals to create awareness; meticulous communicating with transmitting notes between centres created dependability; having the necessary support staff is important; blood bank support and availability of blood products is key to success

		73 patients treated in 29 different community centres	Barriers: smaller hospitals may not have the infrastructure needed to manage leukemia complications—time it takes to get patients to larger center could compromise outcomes; hospital diversion and non-availability of beds at the academic centre can be a frustrating problem for community oncologists
Measures of cost of implementation	No		
**Maintenance: The extent to which program is institutionalized at some duration after intervention (includes reasons for maintenance, discontinuation, or adaptation)**			
Assessed outcomes > 6 months post-intervention	No		
Current status of program	No		
Measures of cost of maintenance	No		
**Law et al. (2021) [21]**			
**RE-AIM Indicators**	Reported? (Yes, No)	Data	Facilitators/Barriers
**Reach: The absolute number, proportion, and representatives of individuals who are willing to participate in CMH shared-care**			
Method to identify patients	Yes	Patients are referred to 1 of 3 regional leukemia centres through an urgent phone call	Facilitators: developing specific criteria for recommending treatment regimens (high-intensity vs. low-intensity)
Inclusion criteria	Yes	Patient's case was reviewed by local hematologist and leukemia specialist to determine eligibility for induction vs. less-intensive therapy	
Exclusion criteria	No		
Sample size and participation rate	Yes	All patients underwent regionalization n= 249 (135 received regional care and 114 received local care alone); participation rate—NR	
Characteristics of both participation and non-participation	Yes	Baseline characteristics described including age, sex, race.	
**Effectiveness: The impact of CMH shared-care on important outcomes**			
Measures/results (at shortest assessment)	Yes	Compared patients diagnosed in 2013–2014 with patients in 2016–2017 after regionalization. After regionalization—more patients received induction therapy (intensive and less-intensive inductions)	

		with implementation of regionalization (65.2% vs. 49%). Also observed reductions in 60-day (HR = 0.67) and 180-day mortality (HR = 0.64)<br><br>Treatment effectiveness was not compared between the groups—focus was on types of therapy received.	
Intent-to-treat analysis utilized	No		
Quality of life outcomes	No		Facilitator: Evaluating patient preferences and satisfaction would be ideal
Percent attrition	No		
**Adoption: The absolute number, proportion, and representativeness of settings, and people who deliver CMH shared-care who are willing to initiate the program**			
Description of intervention location	Yes	Regional centre details described; local centers not described	
Description of staff who delivered intervention	Yes	Regional centres included leukemia physician, hospitalist, nurse, pharmacist, SO, PCC, and clinical educator	
Method to identify staff who delivered intervention (target delivery agent)	No		
Level of staff expertise	Yes	Regional centre staff "experience caring for acutely ill AML patients", no description of local centre	Facilitator: high-quality physician conferences
Inclusion/exclusion criteria of delivery agent/setting	No		
Adoption rate	No		
**Implementation: The intervention agents' fidelity to the various elements of an intervention protocol (including implementation strategy)**			
Intervention duration and frequency	No		
Extent shared-care delivered as intended	Yes	135 patients received regional care, and 114 patients received local care.	Facilitators: coordinator of care and follow-up of patients treated at local centre; implementation of telemedicine for long-term follow-up when patients return to local centre (for continuity and survivorship); ongoing collaboration
Measures of cost of implementation	No		
**Maintenance: The extent to which program is institutionalized at some duration after intervention (includes reasons for maintenance, discontinuation, or adaptation)**			

Assessed outcomes > 6 months post-intervention	No	Regionalization began in 2015	
Current status of program	No		
Measures of cost of maintenance	No		
**Lim et al. (2022) [26]**			
**RE-AIM Indicators**	Reported? (Yes, No)	Data	Facilitators/Barriers
**Reach: The absolute number, proportion, and representatives of individuals who are willing to participate in CMH shared-care**			
Method to identify patients	Yes	Patients are referred to hub centre by physician in home country, generally after failure of initial cancer treatments	Facilitator: My T Cell treatment—program to assist connect doctors in spoke countries to treating physicians in hub (creating networking opportunities); provides background information to patients about travel/accommodations, foreign patients seeking healthcare in Singapore; access to funding
Inclusion criteria	Yes	Patient's condition, health, and QOL are assessed to determine suitability, and discussions with patient re: costs, travel arrangements, tests required	Facilitator: supporting the patient's emotional and mental health needs during selection process; early referral for CAR-T planning; patient support programs to coordinate travels, finance, accommodation and other logistics<br><br>Barrier: travel away from home; interaction with other healthcare systems
Exclusion criteria	No		
Sample size and participation rate	No		
Characteristics of both participation and non-participation	No		
**Effectiveness: The impact of CMH shared-care on important outcomes**			
Measures/results (at shortest assessment)	No		
Intent-to-treat analysis utilized	No		
Quality of life outcomes	No		
Percent attrition	No		
**Adoption: The absolute number, proportion, and representativeness of settings, and people who deliver CMH shared-care who are willing to initiate the program**			
Description of intervention location	Yes	Description provided on hub centre, but no specifics about spoke centres	

Description of staff who delivered intervention	Yes	Reported on spoke physician playing a crucial role in maintaining clinician-patient relationship	
Method to identify staff who delivered intervention (target delivery agent)	No		
Level of staff expertise	Yes	Description of physician expertise in hub centre, and SW, nursing, and administrative support, lab technicians	Facilitator: up-skilling of local hematologists, ongoing support and advice from hub country physician as required during post-treatment monitoring is essential for effective cross-border communication; establishing opportunities for education/discussion<br>- Preceptor model for hands-on experience for physicians/nurses<br>- Presentations at local conferences/symposia, webinars<br>- Case round meetings/networks<br>Training of emergency physicians<br><br>Barrier: Establishing expertise requires time, training, and resources
Inclusion/exclusion criteria of delivery agent/setting	No	No description of the facilities at the spoke centres and whether there were requirements to participate in hub and spoke model	
Adoption rate	No		
**Implementation: The intervention agents' fidelity to the various elements of an intervention protocol (including implementation strategy)**			
Intervention duration and frequency	No		
Extent shared-care delivered as intended	Yes	Spoke country provides inquiry about CAR-T, referral, patient selection, wash-out period in spoke country to remove current medications, and then post-treatment monitoring<br><br>Hub country provides final eligibility assessment, apheresis and treatment, short-term follow-up	Facilitator: Effective communication between hub and spoke teams, advice and support readily available from hub team. Building networks/collaboration between all physicians involved.<br><br>Barrier: wash-out period can vary from days to months; establishing treatment facilities requires time, training, and resources

		4 patients were treated in hub and spoke model for CAR-T	
Measures of cost of implementation	No	Cost was mentioned—but not defined	Barrier: Cost of CAR-T therapy for patients, as well as impact on national and global healthcare expenditure
**Maintenance: The extent to which program is institutionalized at some duration after intervention (includes reasons for maintenance, discontinuation, or adaptation)**			
Assessed outcomes >6 months post-intervention	No		
Current status of program	No		
Measures of cost of maintenance	No		
		**Muir et al. (1992) [22]**	
**RE-AIM Indicators**	Reported? (Yes, No)	Data	Facilitators/Barriers
**Reach: The absolute number, proportion, and representatives of individuals who are willing to participate in CMH shared-care**			
Method to identify patients	No	Process to identify patients not described.	
Inclusion criteria	Yes	Determined by geographic location; no description about the patients who chose not to participate	
Exclusion criteria	Yes	Described 45 patients not in the region, thus not included. 10 patients that did not achieve remission, thus were not eligible.	
Sample size and participation rate	Yes	191 were referred to Regional centre (45 patients were residents within the region, thus not included) 146 patients were eligible for shared-care—59 participated (participation rate 40%);	
Characteristics of both participation and non-participation	No		
**Effectiveness: The impact of CMH shared-care on important outcomes**			
Measures/results (at shortest assessment)	Yes	When age-matched with comparison group, the 49 patients achieving remission, management by shared-care produced similar survival rates to those treated entirely at regional centre.	Barrier: Not feasible to compare rigorously the survival of shared-care and non-shared-care, potential bias as the regional centre likely retains more difficult cases with worse prognosis

		Regional centre workload is reduced (not measured), enabling the specialist clinicians, nursing, and facilities to devote time to care for patients requiring more demanding therapy, individually tailored treatment strategies	
Intent-to-treat analysis utilized	Yes		
Quality of life outcomes	No		
Percent attrition	Yes	10 patients were excluded as they did not achieve remission with induction (no longer eligible)	
**Adoption: The absolute number, proportion, and representativeness of settings, and people who deliver CMH shared-care who are willing to initiate the program**			
Description of intervention location	Yes	Described regional centre, but did not include details of local hospitals	
Description of staff who delivered intervention	No		
Method to identify staff who delivered intervention (target delivery agent)	No		
Level of staff expertise	No		
Inclusion/exclusion criteria of delivery agent/setting	No		
Adoption rate	No		
**Implementation: The intervention agents' fidelity to the various elements of an intervention protocol (including implementation strategy)**			
Intervention duration and frequency	No		
Extent shared-care delivered as intended	Yes	Induction at specialist centre, with maintenance therapy provided at local centre.	Facilitator: referring centres are happy to treat in co-operation with specialist in the knowledge there is a 'back-up' service if needed.<br><br>Clearly defined chemotherapy protocols.
Measures of cost of implementation	No		Barrier: Referring hospitals may require appropriate funding (which could be taken from the regional centre's funds)
**Maintenance: The extent to which program is institutionalized at some duration after intervention (includes reasons for maintenance, discontinuation, or adaptation)**			
Assessed outcomes >6 months post-intervention	No		

Current status of program	No		
Measures of cost of maintenance	No		
**Slater et al. (2022) [24]**			
**RE-AIM Indicators**	Reported? (Yes, No)	Data	Facilitators/Barriers
**Reach: The absolute number, proportion, and representatives of individuals who are willing to participate in CMH shared-care**			
Method to identify patients	No		Facilitator: Regional case managers help to build trust with patients/families
Inclusion criteria	No		
Exclusion criteria	No		
Sample size and participation rate	No		
Characteristics of both participation and non-participation	No		
**Effectiveness: The impact of CMH shared-care on important outcomes**			
Measures/results (at shortest assessment)	No	No measurements reported on the outcomes of CMH shared-care<br><br>Qualitative outcomes on role of regional case managers:<br>role description, patient advocacy, staff and patient education, sharing information, family support and coordination of care, and facilitators for appropriate environment and multidisciplinary teamwork.<br><br>Growth of pediatric services in SCUs allowed for more specialized care to be provided (not measured); patients received faster treatment in a quieter SCU environment.	Facilitator: access to easy and cheaper parking options.
Intent-to-treat analysis utilized	No		

Quality of life outcomes	Yes	RCM had reported that families appreciated they could have safe care close to home with support of family, school, and local community	
Percent attrition	No		
**Adoption: The absolute number, proportion, and representativeness of settings, and people who deliver CMH shared-care who are willing to initiate the program**			
Description of intervention location	Yes	Described facilities of SCUs including critical care areas, ER etc.	Facilitator: reassuring to families when SCUs had same clinical equipment as tertiary centre; an app was developed with contact details and navigation to the SCUs.
Description of staff who delivered intervention	Yes	Description of the regional case manager role to provide care coordination and treatment under leadership of local pediatrician and in collaboration with tertiary centre. Study evaluated measures related to effectiveness (value and outcomes) of regional case managers including attributes, knowledge, and experience required.<br><br>Description of multidisciplinary team—physicians, SW, psychology, music therapy, PT, pharmacy	Facilitator: regional case managers facilitated shared-care by:<br>- Providing specialised knowledge and experience in clinical care of patients<br>- Continuity of care for patients<br>- Maintaining communication—sharing information<br>- Program leadership<br>- Education to patients/families and other staff<br>- Administrative—activity reporting and risk management<br>- Advocating (for additional nursing positions, representation on hospital committees)<br>- Care coordination including arranging travel to tertiary centre.<br><br>Facilitator: dedication pharmacist to improve safety at SCUs; designated lead pediatricians allowed for continuity for patients; monthly video conferences between case managers and tertiary centre—to discuss issues, complex cases, journal articles, feedback from families.
Method to identify staff who delivered intervention (target delivery agent)	Yes	Described implementation of regional case manager role	
Level of staff expertise	Yes	Discussed various competencies for nurses (including central venous catheter care, chemotherapy administration)	Facilitator: 24 h hotline for support; workshops for pediatricians to discuss complex cases, and receive updates on new treatments and clinical trials; helpful to have a

			dedicated RCM to care for this small group of patients requiring specialized care.<br><br>Barrier: Some regional case managers worked part time—challenge to maintain competency.
Inclusion/exclusion criteria of delivery agent/setting	Yes	Discussed the possibility of patients holidaying around the state and being able to go to other SCUs within the network	Facilitator: RCM's had developed a network of specialized care.
Adoption rate	No		
**Implementation: The intervention agents' fidelity to the various elements of an intervention protocol (including implementation strategy)**			
Intervention duration and frequency	No		
Extent shared-care delivered as intended	Yes	Description of various clinical care provided at shared-care units including direct care with chemotherapy, maintaining communication (EMR, phone, email), patient education, support for patients, administration—patient documentation.	Facilitator: Creation of a network to provide governance and support of a shared-care model (informs decision making at service delivery and corporate levels, provides advocacy, development of guidelines, commitment to delivery of safe care, oversight of QI initiatives, research, education, coordination, project management, implementation—including information resources and education); software to share information and monitor progress; development of forms for pre-chemotherapy assessment, chemo administration, febrile neutropenia pathways.<br><br>Facilitator: implementation of electronic medical records – accessible information; smaller volumes of patients than tertiary centre helped SCU's devote more time to working with families<br><br>Barrier: Lack of designated area for treatment of patients at SCUs; electronic record sharing reduced phone call communication (e.g., Mum's a bit fragile today).
Measures of cost of implementation	No		
**Maintenance: The extent to which program is institutionalized at some duration after intervention (includes reasons for maintenance, discontinuation, or adaptation)**			

Assessed outcomes >6 months post-intervention	No	Established in 2006
Current status of program	No	
Measures of cost of maintenance	No	
